# Supplementary material for: Analysis of the Conformational Landscape of the N-Domains of the AAA ATPase p97: Disentangling the Continuous Conformational Variability in Partially Symmetrical Complexes
Source: Int J Mol Sci. 2024 Mar 16;25(6):3371. doi: 10.3390/ijms25063371 (PMC10970294; doi:10.3390/ijms25063371)
Supplement: Supplementary file 1 [file ijms-25-03371-s001.zip › ijms-2800745-supplementary.pdf]

Supplementary Materials

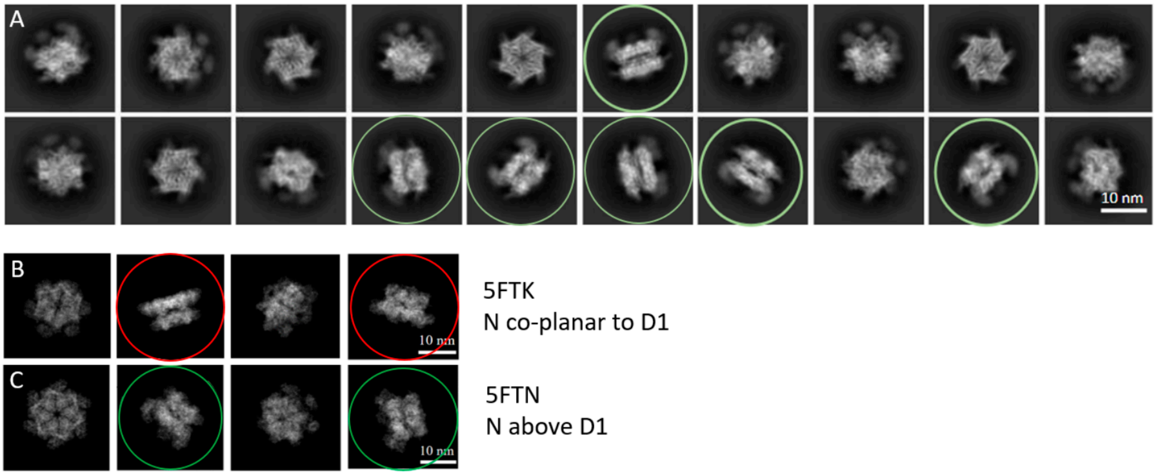

Figure S1. 2D classification of p97R155P-ATPγS. (**A**) Representative main classes obtained after removing false positive from the automatic particle picking. These classes comprise the 274,640 particles analyzed in this study. The N-domains position above the D1 ring as seen in the side views (circled light green). (**B**,**C**) Simulated projection images of the models of p97 with the N-domains in the co-planar conformation (pdb id: 5FTK, side views encircled red) and the N-domains in the up conformation (pdb id: 5FTN, side views encircled dark green).

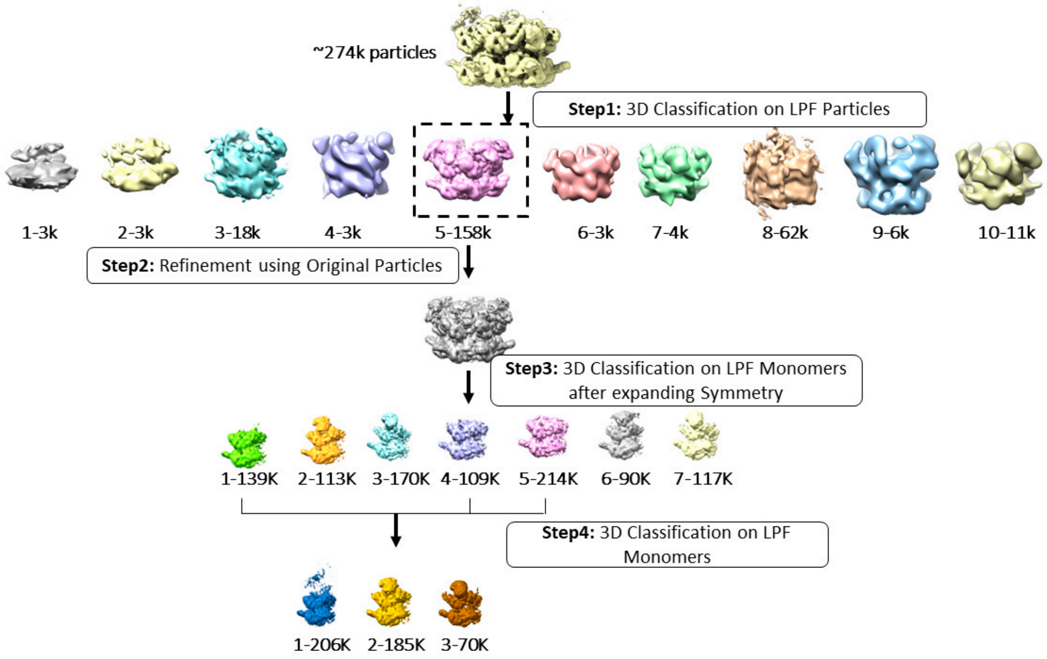

Figure S2. Overall processing steps of the focused classification using low-pass filtered images. Particle images were low-pass filtered (LPF) using a 6Å cut-off, a resolution at which alpha helices should still be visible. A first classification using the complete signal for the hexamer was performed. A second classification was performed focusing on the signal of a monomer, after pre-processing the images using the C6 symmetry expansion and signal subtraction of 5 monomers as described in Figure 2.

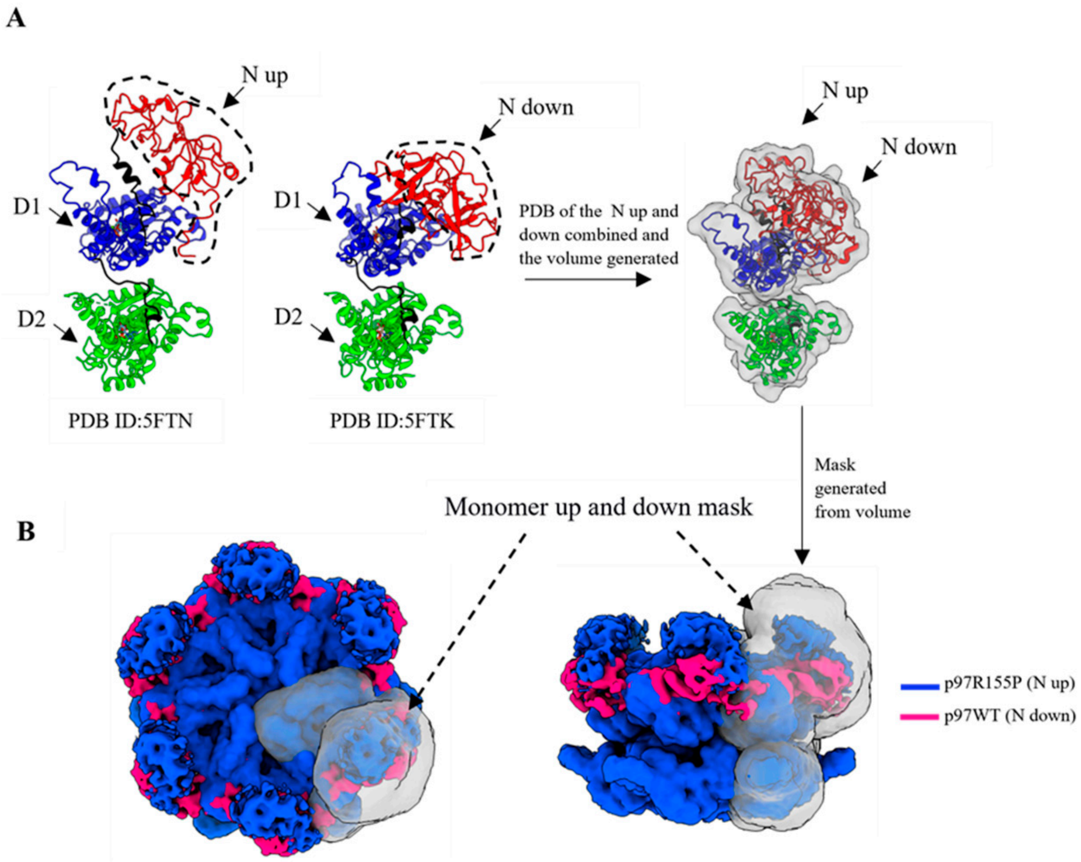

Figure S3. Generating a mask for monomers with the N domains in the up and down positions. (**A**) The volume generated from the combined PDB file of N up (PDB ID: 5FTN) and N down (PDB ID: 5FTK) in Chimera. The N, D1 and D2 domains are colored red, blue, and green. The generated volume is shown as transparent. (**B**) The generated volume from PDB file was used to create a mask in Relion. The monomer up and down mask is shown as transparent on the superimposed map of p97WT-ATPγs (pink) and R155P-ATPγs (blue) map.
